# Identifying context-specific drivers of routine childhood immunisation dropout in Mozambique and Malawi: a secondary thematic analysis of qualitative community-based participatory research data

**DOI:** 10.1136/bmjopen-2025-104490

**Published:** 2025-11-19

**Authors:** Emily Lawrence, Alina Metje, Charles Matemba, Jocelyn Powelson

**Affiliations:** 1 VillageReach, Seattle, Washington, USA; 2 VillageReach, Lilongwe, Malawi

**Keywords:** Implementation Science, PUBLIC HEALTH, QUALITATIVE RESEARCH, Community-Based Participatory Research, Immunization Programs

## Abstract

**Abstract:**

**Objective:**

Routine childhood immunisation is vital to preventing life-threatening illness; however, global coverage of routine childhood immunisations has fallen in recent years, leaving over 14 million children globally without protection. This study aimed to identify shared and context-specific drivers of routine childhood immunisation dropout in select sites in Mozambique and Malawi through a secondary analysis of qualitative data.

**Design:**

We conducted a secondary inductive thematic analysis on qualitative data from a community-based participatory research study. Co-creation workshops, guided by a human-centred design approach, were held to develop context-specific solutions in each study site. Data for this analysis were collected between February 2020 and March 2021 in Mozambique and between July 2022 and February 2023 in Malawi.

**Setting:**

Zambezia, Mozambique and Lilongwe and Mzimba North Districts, Malawi.

**Participants:**

Participants included caregivers of partially (n=60) and fully vaccinated (n=22) children aged 25–34 months, healthcare workers (n=12), community healthcare workers (n=30), Expanded Programme on Immunisation staff (n=11) and community representatives (n=14). Caregivers were identified through vaccination registers and with support from health workers, community leaders and health volunteers.

**Results:**

We identified four key contextual and health system differences between Malawi and Mozambique affecting dropout: the composition of the immunisation workforce, the state of the vaccine ecosystem, vaccination card policies and vaccination outreach models. Common challenges across both countries included gender roles that burdened mothers, limited vaccine information, negative health worker interactions, pandemic-related disruptions and stockouts. Common solutions generated through co-creation workshops included improving health worker–caregiver communication, vaccine education and immunisation outreach resources. Solutions in Mozambique emphasised strengthening the community health worker (CHW) role in immunisation, while in Malawi, whose CHW workforce already administers vaccines, solution ideas focused on improving CHW data management.

**Conclusions:**

Our analysis highlights the opportunity for scalable solutions to identified common immunisation barriers, including tailored vaccine education that addresses caregiver knowledge gaps, improved caregiver–health worker communication, improved outreach models and addressing gender dynamics and vaccine stockouts.

**STRENGTHS AND LIMITATIONS OF THIS STUDY**

- Participatory qualitative methods, such as photovoice, photo elicitation and short message exchanges empowered participants to share detailed and nuanced descriptions of their experiences with routine childhood immunisation and built rapport and trust with caregivers.
- Selection bias may have occurred due to inconsistent recruitment methods between study sites, differences in vaccination registry practices and difficulties in recruiting participants from specific demographic groups such as migrant populations, tenant farmers and specific religious communities.
- Data in Mozambique and Malawi were collected 2 years apart; although this was considered during analysis and is reflected in our findings, the time difference between data collection may have impacted the ability to make a direct comparison between the two study sites.

## Introduction

 Routine childhood immunisation is a cornerstone of public health, protecting children from a multitude of life-threatening preventable diseases. Despite its crucial role, gains in global routine immunisation coverage began to slow from 2010 to 2019.^1^ Global childhood immunisation coverage has still not recovered from the COVID-19 pandemic, which severely disrupted global vaccine supply chains and routine health services.^2 3^ In 2023, an additional 2.7 million children were unvaccinated or undervaccinated compared with prepandemic levels in 2019, and a staggering 14.5 million children were considered ‘zero dose,’ meaning they did not receive their first dose of the diphtheria-pertussis-tetanus vaccine.^4 5^ This trend underscores the urgent need for renewed efforts to enhance immunisation programmes.

Current literature highlights numerous barriers to routine immunisation uptake, such as poor knowledge about vaccine schedules, limited access to health facilities, fear of side effects and negative service experience.^6^,^9^ However, there is limited understanding of the context-specific health system and contextual factors influencing these barriers, especially in diverse geographical and health system settings. A critical gap remains in understanding how these factors vary between and across countries and how these variations impact routine immunisation experiences and outcomes, and in exploring potential solutions that could work across different contexts. This issue is particularly pressing in Malawi and Mozambique, where subnational variations in geographic, cultural and socioeconomic contexts have led to inconsistent immunisation rates across different regions.^10 11^

We conducted a secondary cross-country analysis using qualitative data to compare the drivers of under-2 routine immunisation dropouts and identify potential solutions within and across diverse contexts in Malawi and Mozambique. The original studies explored drivers of under-two immunisation dropouts within each context and used the same theoretical framework, similar tools adjusted slightly to context, and similar data collection and analysis methods across both study sites.^12 13^ This secondary analysis aims to highlight commonalities and unique contextual and health system factors that may impact routine immunisation dropouts.

## Methods

### Study site contexts

#### Malawi

Immunisation has been a high priority for Malawi’s Ministry of Health since the establishment of the Expanded Programme on Immunisation (EPI) in Malawi in 1979.^14^ Routine vaccine services are provided at a combination of public static and outreach sites as well as private facilities, which administer an estimated 27% of vaccines in Malawi.^15^ Children are also vaccinated through large-scale campaigns to deliver high-priority vaccines such as the polio vaccine or during disease outbreaks such as cholera. While vaccines at all facilities are provided free of charge by the government, there may be additional fees associated with other aspects of the vaccination process, for instance for health cards or weighing the child.^15^ Across all facilities and service delivery methods, vaccines are administered by Malawi’s cadre of community health workers (CHWs), called Health Surveillance Assistants, who are also responsible for extending basic health services into the community.^16^ Contrary to coverage trends in most countries, vaccination coverage in Malawi’s rural communities is higher than in urban areas.^11^ While routine immunisation coverage in Malawi is relatively high compared with surrounding countries, with over 80% coverage for most vaccines, coverage rates have plateaued or even declined over the past decade and threaten to continue doing so in the wake of COVID.^5 17^ Dropout from the first dose of the DTP vaccine to the last routine dose of the measles containing vaccine (MCV) is estimated at 27%, pointing to the need for continued efforts to strengthen vaccination systems in the country.^18^

#### Mozambique

Routine immunisation services in Mozambique are provided almost exclusively by the public sector through a combination of fixed health posts and outreach services, called mobile brigades. Door-to-door campaigns are also conducted for specific vaccines such as polio or measles. Vaccines are administered by trained health workers who balance their roles as vaccinators with their other primary healthcare (PHC) responsibilities.^19^ The CHW cadre of Agentes Polivalentes de Saúde supports vaccination efforts by providing immunisation education in the community and assisting at mobile brigades, but they do not routinely administer vaccines.^20^ Access to health facilities in Mozambique remains a challenge for many communities; more than half of the population lives an hour or more away by foot from the nearest health facility.^21^ Mobile brigades are recognised as an essential strategy for bringing vaccines to those populations, but these outreach efforts are hindered by resource constraints; in 2017, mobile brigades were responsible for only 10% of all routine vaccines delivered in the country. Mozambique’s immunisation coverage was heavily impacted by COVID-19, with coverage of the third dose of DTP fluctuating from 85% in 2019 to 55% in 2020, and most recently jumping back to 70% in 2023.^22^

Table 1 summarises the key characteristics of the immunisation systems in Malawi and Mozambique.

**Table 1:** Immunisation system characteristics—Malawi and Mozambique

	Malawi	Mozambique
Primary healthcare (PHC) services	Services are delivered through a combination of public facilities (48% of services), private for-profit facilities (24% of services) and private not-for-profit facilities including the Christian Health Association of Malawi (29% of services). Basic PHC services are extended into the community through outreach efforts delivered by CHWs and volunteers.^41^	The vast majority of PHC services are delivered through public facilities. Infrastructure is limited, and over half of the population lives an hour or more by foot from the nearest facility. CHWs are typically located in harder-to-reach communities more than 8 km from the nearest facility and provide basic health services, not including immunisation.^20 21^
Immuniser cadre	Routine immunisation across public and private sites is largely provided by CHWs. In addition to administering vaccines, CHWs are also responsible for vaccine education and record-keeping and for providing basic health services in the community. The country faces a shortage of 7000 CHWs, with most districts having fewer than 70% of CHW positions filled.^11 34^	Routine immunisation is provided by health workers and immunisation technicians. This cadre is also responsible for providing other facility-level PHC services. CHWs play a role in vaccine education and community mobilisation but do not deliver vaccines themselves.
Outreach services	Malawi’s strategy to reach every child with vaccination services relies heavily on vaccination outreach sessions in which CHWs travel to specific community sites to deliver vaccines. In 2016, a total of 4894 outreach clinics were conducted by 781 static facilities.^42^	Outreach services in Mozambique are delivered through mobile brigades. Mobile bridges are supposed to be conducted quarterly in all communities that reside further than 8 km from the nearest facility, but services are disrupted by resource constraints and other logistical challenges.^19^
Vaccine ecosystem	In the past decade, Malawi has introduced multiple new vaccines to the routine immunisation schedule, including the second dose of MCV in 2015, the malaria vaccine in 2019 and the typhoid conjugate vaccine in 2023. In the last 5 years, widespread vaccination efforts were conducted against COVID-19, typhoid, measles, rubella, polio and cholera.^43^,^46^	Mozambique added the rotavirus vaccine and second dose of measles-containing vaccine to the routine immunisation schedule in 2015. The malaria vaccine was added to the schedule in 2024. In the last 5 years, widespread vaccination efforts were conducted against COVID-19, polio, measles, rubella and cholera.^47^

CHW, community health worker; MCV, measles containing vaccine; PHC, primary healthcare.

### Study design

We conducted a secondary analysis of qualitative data on drivers of routine immunisation dropout collected using community-based participatory research and human-centred design (HCD) approaches in select sites in Mozambique and Malawi.^12 13 23 24^ This secondary analysis aimed to answer the following questions:

- How do context-specific factors contribute to similarities and differences between drivers of under-2 immunisation dropouts between Malawi and Mozambique study sites?
- How do context-specific factors contribute to similarities and differences between potential solutions to address under-2 immunisation dropouts identified through the HCD process between Malawi and Mozambique study sites?

An initial grant supported research from February 2020 to March 2021 in Gile and Namarroi Districts in Zambézia Province, Mozambique. A follow-on grant then expanded this research to Lilongwe and Mzimba North Districts in Malawi from July 2022 to February 2023. The second grant expanded the scope of the study in three ways: it added an urban site to enable urban–rural comparisons; supported cross-country analyses of similarities and differences between findings in Malawi and Mozambique; and examined which interventions could be scaled broadly versus which required context-specific adaptation to strengthen full immunisation coverage.

In both Mozambique and Malawi study sites, we hired and trained four female caregivers of children 36 months or younger as coresearchers to provide input on the study design, collect all qualitative data, participate in the primary analysis to identify drivers of under-2 immunisation dropouts and co-facilitate the HCD workshops. These caregiver researchers were selected based on their familiarity with the study communities, fluency in local languages, prior research experience and experience navigating the immunisation system with their own children. Caregiver researchers participated in an in-person 5day training on qualitative methods, research ethics and interview techniques, as well as short refresher training throughout the study period.

The qualitative data collection tools were structured around the WHO’s Behavioural and Social Drivers of vaccination framework and the UNICEF Immunisation Journey framework.^25^ UNICEF’s HCD for Health guide was leveraged for the HCD cocreation workshops in which participants validated study findings and then ideated and developed tailored solutions to identified barriers.^26^

The research team consisted of local, national and international researchers and practitioners with diverse disciplinary, linguistic and cultural backgrounds. To mitigate bias and strengthen reflexivity, all researchers participated in regular debriefs during data collection and analysis. These sessions provided the opportunity to reflect on how their lived experiences and professional backgrounds may be shaping the ways data were collected, interpreted and analysed. Data interpretation was done collaboratively, and to help ensure the validity of the findings, preliminary results were shared with participating health facilities and community representatives for feedback and validation.

### Study setting, population and recruitment

In collaboration with Malawi and Mozambique Expanded Immunisation Programme (EPI) staff, we selected 8 health facilities in each country, four per district, based on high dropout rates, diverse geographical representation and accessibility for data collection. Participants included caregivers of fully and partially vaccinated children and health staff who administer routine immunisations. Caregivers were defined as parents or guardians who took PHC seeking responsibility for a child. Caregivers were eligible if their child was 25–34 months old, an age by which they should have completed the under-2 routine immunisations while still being young enough to minimise potential recall bias. Vaccination status was determined by whether the child had completed all recommended doses on the respective country’s immunisation schedule by age two. Eligibility was initially determined by examining the health facility records and then confirmed by the child’s vaccination card. Vaccination status was determined according to the vaccination card in cases where there were discrepancies with health facility records. If the immunisation card was unavailable, caregivers were asked to describe which vaccines their children had received and were excluded if they were uncertain of their child’s vaccination status.

Health workers in Mozambique and CHWs in Malawi were purposively recruited from the same 16 health facilities based on their roles in administering and managing routine immunisations. No health workers or CHWs declined to participate in the study.

Sample sizes of caregivers, health workers and CHWs were determined based on feasibility, taking into consideration anticipated recruitment challenges and travel requirements, as well as based on the need to capture the diversity of experiences of participants (vaccination status, facility type, etc). During data collection, we assessed progress towards thematic saturation, which in Malawi led to increasing the sample to an additional 10 caregivers to reach saturation.^27^

We held six co-creation workshops, three in Malawi and three in Mozambique. The first four workshops were ideation workshops held in each study district, where participants reviewed the results on the drivers of under-2 immunisation dropout for their study sites and then ideated how to address those barriers through a series of participatory activities. Participatory activities included: reviewing personal profiles, journey maps and key findings; crafting problem statements, conducting a strengths, weaknesses and threats analysis, rotating idea centres and implementation versus feasibility matrices. Ideas were then presented to respective EPI representatives in Zambezia and National EPI in Malawi to select the top ideas to develop further during solution prototyping workshops. EPI representatives selected top ideas based on the following criteria: alignment with government priorities, feasibility to implement in 1 year and with a budget of US$75 000 in Mozambique and US$50 000 in Malawi and potential for impact. Once the top ideas were chosen, a second workshop was conducted to finalise the solutions and co-create a prototype. Participants in the HCD workshops included a subsample of caregivers, health workers and CHWs who participated in the data collection phase, EPI representatives from the district and provincial level (Mozambique) and national level (Malawi), as well as selected community leaders and community representatives from the original study sites. Caregivers were selected for participation based on whether they had expressed interest during the data collection phase and their accessibility to the workshop location; community members, CHWs and health workers were purposively selected based on accessibility to the workshop location.

### Data collection

Semistructured interviews supplemented with photovoice in Mozambique and photo-elicitation in Malawi were used to document caregivers’ experiences with the under-2 immunisation process and to identify factors associated with completion or dropout of the routine immunisation schedule.^28 29^ In Mozambique, for photovoice, caregiver researchers provided digital cameras to caregiver participants, gave instructions and demonstration on their use, and asked caregivers to take photos related to their child’s immunisation experience. Caregiver researchers returned 2–6 days later to ask the participants to select the top five photos that they felt best represented their experience and to explain the meaning and significance of each selected photo as it related to their child’s immunisation experience. A semistructured interview followed the photo discussion. In Malawi, photo-elicitation interviews were conducted by showing caregivers a set of 37 photos that depicted different vaccination processes, barriers and facilitators. Caregiver researchers started each interview asking caregivers to think back on their child’s immunisation experience and to then look at the photos and pick up to five photos that best depicted their journey. Caregiver researchers then discussed each of the selected photos to understand how it related to their journey and followed with a semistructured interview. Interviews were conducted in the caregiver’s language of preference, either Tumbuka, Chichewa or Elomwé.

Health workers in Mozambique and CHWs in Malawi participated in message exchanges and semistructured interviews in the language of preference (Tumbuka, Chichewa, Portuguese or English) about their perceptions of caregiver routine immunisation experiences, beliefs about causes of routine immunisation dropout and their experience administering vaccines to children under 2. For the message exchanges, health workers and CHWs were provided with phones with a Telegram group chat preset on the phone to facilitate the message exchanges. Over a 3-week period, caregiver researchers prompted health workers and CHWs to send messages or photos related to notable routine immunisation experiences that week and then followed up with probing questions. At the end of the 3 weeks caregiver researchers conducted a semistructured interview with each participant.

In Malawi, in addition to the two methods above, we also conducted 16 observations of immunisation sessions at health facilities and outreach sites across the eight study sites. These observation sessions lasted approximately 4 hours, during which caregiver researchers took notes about the immunisation processes, attendees and attitudes and behaviours of CHWs.

### Data analysis

All interviews were audio-recorded, translated and transcribed by caregiver researchers into English in Malawi and Portuguese in Mozambique. In Mozambique, transcripts were additionally translated into English for analysis. A team of three researchers conducted the secondary analysis of the transcripts. All transcripts were imported into ATLAS.ti. (V.23) to support data management and analysis. An initial codebook was developed after open coding 11 caregiver, health worker and CHW interviews from Mozambique and Malawi.^30^ The remainder of the transcripts was then coded, adding new codes as needed. All codes were summarised by country to facilitate a cross-site inductive thematic analysis. Lastly, the main themes and comparisons between findings from the Malawi and Mozambique study sites were elaborated.

### Patient and public involvement

The caregiver researchers, as representatives of the study population, participated in the collection and analysis of the primary data on which this secondary analysis was based.

## Results

### Participants

Table 2 summarises the number of study participants by country and participant type.

**Table 2:** Study participants

Participant type	Mozambique (N)	Malawi (N)
Caregiver of a fully vaccinated child	10	12
Caregiver of a partially vaccinated child	22	38
Healthcare worker	12	0
Community healthcare worker	0	30
Community leaders	4	0
EPI representatives (human centred design workshops)	4	6
Community group representatives (human-centred design workshops)	0	3
Partner organisation representatives (human centred design workshops)	0	3
Senior community healthcare worker (human-centred design workshops)	0	4

EPI, Expanded Programme on Immunisation.

Online supplemental table S1 summarises the key findings presented below.

### Factors that may drive differences in immunisation experience

The following themes present contextual or health system factors that contribute to differences in routine immunisation experiences and routine immunisation dropout between Malawi and Mozambique.

#### Composition of the immunisation health workforce

In Mozambique, health workers frequently described feeling overworked, understaffed and burdened with responsibilities beyond immunisation during vaccination sessions. As one health worker in Mozambique described *“…in the remote health facilities we use the single door strategy where all activities are offered by the same provider/technician…*”. High turnover among health workers was a common concern, with some participants noting that some facilities went months without a staffed health worker or vaccination services. This lack of continuity in healthcare workers (HCWs) and in some cases, pauses in access to care and vaccination services, eroded caregivers’ trust in immunisation services and prevented them from getting their child immunised.

… I don’t trust [the nurse who vaccinated my child] because of the changes, this is because they bring a nurse who after some time we get used to, we even like their work, and then they are transferred, in their place comes another nurse who is often impatient, to get used to the new one takes a long time and gets very complicated for us. – Mozambique, Caregiver

In contrast, CHWs in Malawi described generally well-staffed vaccination sessions and that duties during sessions were divided between teams. They also described leveraging community volunteers to assist with tasks like weighing children and record keeping.

In outreach, there are 4 health workers. There are two injectors. One writes the register and the other provides family planning. On the weighing scale, there are volunteers from the community.*-* Malawi, CHW

While staffing concerns were present in Malawi, particularly for outreach services, during new vaccine introductions or campaigns, they were less pronounced. Unlike Mozambique, turnover was not mentioned, and caregivers in Malawi generally recognised CHWs as consistent, trusted community members who also contribute to other tasks in the community, as one caregiver in Malawi described, *“I trusted them [CHWs] because they are the ones who vaccinated my first-born child.”*

#### State of the vaccine ecosystem

At the time of data collection, Malawi was undergoing multiple vaccination campaigns including COVID-19, cholera and polio, in addition to trials for the malaria vaccine. As a result, participants in Malawi cited confusion about the different vaccination campaigns and how these related to routine immunisations, with some fear of children getting too many vaccinations or experiencing unwanted side effects.

We give the polio vaccine during the campaign… when we give it to people in that village, we go door to door telling them that this polio vaccine prevents paralysis. When they hears [sic] that information…they reply that he already received it… so I told them ‘No, that one is from the campaign, this one is from the hospital here’… but the people, they don’t understand, they think that there are too many vaccinations, they say that you want this vaccination so that our children should be infertile in future.- Malawi, CHW

Specifically, the introduction of the COVID-19 vaccine in study sites in Malawi led to decreased trust in vaccines in general, with CHWs citing circulating rumours about the vaccine and some caregivers citing fears that some routine vaccinations were being replaced with the COVID-19 vaccine.

…the doubts may come in when some people think that our child may receive Covid 19 vaccine instead of children’s vaccine and this is why most women have doubts to vaccinate their children that maybe their children may receive Covid 19 vaccine. – Malawi, Caregiver

Overall, the combination of these events led to confusion and an environment of increased vaccine hesitancy among some caregivers and their communities in Malawi.

In Mozambique, these concerns were less evident. At the time of data collection, the COVID-19 vaccine had not yet been introduced in the study sites and there were no concurrent outbreaks or emergency campaigns. However, in both countries, the recent introduction of the second measles dose (MCV2) caused confusion, with many unaware that their child needed a second dose of measles, believing the vaccine schedule ended after 9 months.

…most of the times when mothers are given that 9-month vaccine, they hardly ever come back to comply with the second dose of measles vaccine, which is the vaccine that we administer at 18 months, perhaps because the old calendar the mother just had to complete the 9-month vaccine… - Mozambique, HCW

#### Vaccination card policies and practices

Vaccination cards were universally recognised as essential tools to remember when vaccinations were due and were often required to receive vaccinations in both countries. However, HW and CHW responses to lost cards varied significantly, impacting caregivers’ experiences and their children’s immunisation outcomes.

In Mozambique, caregivers commonly reported being denied vaccines in the event they lost or damaged the card. For example, one caregiver noted:

…when we arrive at the health facility… we stay in the queue with the health card of the child in our hand so that we can get vaccinated…we always take the child’s health card, because the medical technicians don’t take care of us without the card…one does not forget it [the vaccination card]*.-* Mozambique, Caregiver

In Malawi urban study sites, caregivers without an immunisation card reported that they were required to wait longer and at times were served last at immunisation sessions. Health facilities in these urban sites reported not keeping vaccination registers and instead relied on caregiver recall to recreate records and, in some instances, restart the child’s vaccination schedule. As a result, some caregivers feared returning to the facility without a card, anticipating their child might receive repeated vaccines.

[if there is a missing vaccination card] we rely on information from the mother, if the mother remembers clearly,because others can describe the month of, on the date that he was injected here and there… will continue with the vaccinations that have not been given or the ones that are left, but if the woman is not giving pictures of anything tangible, regarding this vaccine, we have no choice but to start her again. - Malawi (urban), CHW

In Malawi rural settings, unlike Mozambique and urban Malawi sites, a missing vaccination card was not mentioned as a reason for missed vaccination. Respondents reported when a card was missing, health workers would check the registry books to see which vaccines the child already received and would write them on a new vaccination card. If the caregiver could not afford a new card, CHWs sometimes reported writing it on a piece of paper.

#### Vaccination outreach model

Across both Mozambique and Malawi, caregivers expressed the long distances to vaccination sites, lack of health facilities in their areas and/or significant time spent getting to sites as key barriers to being able to complete their child’s routine immunisations.

I want to share the suffering that we have every day in covering a long distance, when we took our children to the hospital to vaccinate… Every time I take my son to the hospital I come back with pain and swelling in my legs and feet, which is one of the reasons why I don’t come back the following month, since I have no money to pay for transportation, nor anyone to help me*…* – Mozambique, Caregiver

In both countries, caregivers noted that efforts that brought vaccines closer to them, like mobile brigades in Mozambique and outreach sites and door-to-door follow-up in Malawi, made vaccination easier and more accessible.

In Malawi, CHWs and caregivers cited various ways access to public immunisation services can be accessed, including at the health facility, set outreach sites and in some cases home visits. Additionally, in Malawi, CHWs leveraged mother care groups and home visits to follow-up with children who were behind on vaccines. Outreach services in Malawi were reported as more reliable and frequent, with caregivers having knowledge of when and where outreach sessions were taking place and able to rely on them as a place to get their child vaccinated.

…so, usually I get my child’s vaccine at our outreach clinic so for some who are closer to the health center they go there but for me since I am close to this outreach clinic this is where I go to for my child’s vaccines so when we go there they give us like some dates when we ar*e* supposed to go and get them vaccinated *–* Malawi, Caregiver

In comparison, in Mozambique, opportunities for mobile brigades were limited and poorly coordinated, and caregivers were often unaware of their timing or location. In Mozambique sites, there was no mention of alternative outreach approaches, and most caregivers and health workers described receiving/providing vaccinations at the static health facility.

One of the main factors that causes dropouts is the absence of mobile brigades *–* Mozambique, HCW

##### Identified factors that may drive similarities in immunisation experience

Despite contextual differences, several common challenges emerged across both country study sites.

### Gender roles and family dynamics

In both countries, the mothers were considered the primary person responsible for vaccination. If a mother is unwell, overburdened or lacks active support with their other responsibilities, vaccination is often missed. Health workers, CHWs and mothers themselves described one of the causes for missed vaccines as a mother’s ‘laziness’ or ‘negligence’ and failure to uphold caretaking responsibilities, often later citing specific reasons that may have hindered vaccination such as lack of vaccines or a lost health card.

What happened is that I got pregnant before this whole vaccination process was completed. I couldn’t carry the other child, while being pregnant, to the hospital to get vaccinated, because it is not easy to borrow a bicycle without paying and I didn’t have any money, and I ended up giving up. My husband did not help to take me to the hospital to get vaccinated, I only went with him to open the pre-natal record and from then he never helped again. – Mozambique, Caregiver

Their beliefs about vaccinations in my community, there is no problem with vaccine refusal, no, it’s just laziness that women these days have become too lazy. - Malawi, CHW

While some fathers and other family members were involved in decision making around vaccination or, in some cases, across both countries, provided support in the form of transportation to the facility, reminding the mother about upcoming vaccinations or in caring for the child after vaccination, it was not the norm for them to take children for vaccination. There were exceptions, however, in both countries with health workers noting that in some cases this did happen, and when it did, they would often praise the father and encourage the behaviour. When fathers and family members were more actively involved in the immunisation process, it was more likely that the child would receive their needed vaccinations.

He helps me hold the child while I do other household chores, and cook, when the child’s crying persists, I take her while he continues with the cooking *-* Malawi, Caregiver

### Insufficient vaccine information

Caregivers generally held positive views on vaccines and desired for their children to be vaccinated. However, many cited a lack of key information to help guide their decision making and understanding of when to access immunisation services. In both settings, information gaps were primarily around the immunisation schedule and confusion around vaccine safety, particularly when children were catching up on missed vaccines or vaccinated while ill. Some caregivers also expressed concerns about side effects, especially when their child received multiple vaccines at once, including doses beyond what they expected from the standard schedule, such as during campaigns or catch-up visits. This contributed to hesitancy to return to the facility if their child was off schedule.

I did not get the information about the vaccination But the mothers who live near to the hospital receive this information through the lectures that are given earlier before the start of the activities. For those of us who live far from the hospital, we do not have this opportunity to participate in the lecture, even during the vaccination they do not explain anything about the importance and the type of vaccine that the child will receive. – Mozambique, Caregiver

In both countries, there was informal knowledge-sharing between caregivers. Caregivers would sometimes reassure each other about side effects or remind each other about upcoming vaccination appointments. In some cases, these same information networks also resulted in rumours being spread about vaccination.

…my friends influenced my feelings to fully vaccinate my child, they encouraged me a lot, that made me to make a decision to vaccinate my child…At that time, I had questions, if I vaccinate my child what really happens? They were encouraging me that when you vaccinate the child the body changes, he cries too much, because they encouraged and see that it was a good thing then I managed to vaccinate my child. – Malawi, Caregiver

### Negative interactions with health workers

In both countries, caregivers generally expressed trust in health workers; however, some reported being fearful in situations where they had lost a vaccination card, had missed scheduled doses or felt that they or their child was not adequately dressed or bathed. This was often due to having been yelled at themselves or witnessing others being scolded. Across both countries, this fear of being humiliated or yelled at by a health professional deterred them from following up on missed vaccinations.

You know these people [CHWs], they get angry even with any slightest occurrence. So, even though I had questions to ask, I was afraid to ask them questions.- Malawi, Caregiver

In relation to care, it was good despite the insults.I was yelled at by the clinician for not taking the child for vaccination regularly, and this caused me not to return the following month.- Mozambique, Caregiver

### COVID-19 pandemic disruptions

In both countries, the pandemic introduced service disruptions, though the nature and impact on routine immunisation services varied. In Mozambique, where at the time of data collection the COVID-19 pandemic had just started and vaccines were not yet available, mobile brigades were suspended, vaccine supply was inconsistent and fear of infection and lack of resources to prevent infection (masks, hand sanitiser) deterred facility visits. In Malawi, the pandemic had been underway for over a year at the time of data collection and vaccines were available. In both settings, some caregivers reported missing immunisations due to pandemic-related protocols.

“With COVID-19, we stopped doing the mobile brigades, they were interrupted for a certain period of time…” - Mozambique, HCW

### Stockouts and resource constraints

In both countries, health workers expressed higher motivation and job satisfaction when they had all the resources they needed to conduct immunisation sessions. When certain resources were unavailable or when infrastructure was poor, this made it harder for health workers and CHWs to carry out their vaccination responsibilities and added frustration and stress. Resources across both countries tended to be a bigger issue for outreach services (Malawi) and mobile brigades (Mozambique), with health workers and CHWs citing frequent challenges related to availability of transport or fuel to get to outreach/mobile sites. When this happened, outreach services were cancelled. Seasonal weather further exacerbated these resource constraints; during the rainy season, when health workers and CHWs don’t have raincoats or boots or there is no place of shelter at an outreach or mobile site, it makes it harder for them to conduct outreach sessions and is also harder for caregivers to reach static facilities.

[we had a] shortage of safety box and cotton wool and also registers to record people and other records[so there was] there was no progress today [for vaccinations] as work can’t take place without these basic necessities and so there is need to address these issues fast. Malawi, CHW

In both countries, caregivers cited challenges at the health facility such as stockouts of vaccines and long lines for vaccination which negatively impacted their vaccination experience and made them less motivated to return for subsequent vaccine visits.

I could not complete my child’s vaccination because almost every time I went to the hospital, I didn’t find any vaccines, I got frustrated and never went to vaccinate again. *-* Mozambique, Caregiver

#### Similarities and differences in cocreated solutions to address routine immunisation dropout

In co-design workshops in Mozambique and Malawi, participants reflected on the specific results for their study sites and ideated solutions to address the challenges. Across Malawi and Mozambique, similar solution ideas included:

- Improving interpersonal interactions between health workers and caregivers with a focus on cultivating empathy among health workers and CHWs for caregivers, and in Mozambique specifically, training to improve treatment of mothers who come to receive vaccination off schedule.
- Improving the vaccine education provided to caregivers to address specific knowledge gaps such as ensuring that information was provided around the vaccination process and schedule.
- Increasing knowledge and engagement around routine immunisation among men.
- Providing more resources to conduct outreach, such as transport and fuel.
  Differences in solution ideas originating from the workshops related to the roles of CHWs. In Mozambique, participants suggested solutions to increase CHW involvement and capacity in vaccination, such as by having them conduct monthly visits in communities, involving them in outreach and potentially enabling them to vaccinate. In contrast, in Malawi, the focus on CHWs’ role in vaccination was focused specifically on training them on data management so that they are better able to manage vaccine stocks. An additional difference in the workshop outputs between the countries was related to outreach services; participants in Mozambique suggested solutions to improve mobile brigades by increasing their frequency and ensuring that the community is notified as well as by creating committees to carry out home visits, while solutions developed in Malawi focused on improving outreach services more generally, like having CHWs arrive on time and having a covered space to conduct outreach services.

## Discussion

The health system and social factors identified through this analysis are consistent with other studies, but we provide a new perspective by considering how contextual factors like the health system, social structures and norms may influence differences and similarities in immunisation experiences and outcomes.^931^,^33^ This is important when considering what kind of interventions may work in different contexts and the relative ‘fit’ and scalability of a given routine immunisation intervention. Our results further show that even when a solution is scalable and fits the implementation context, it is still important to tailor solutions based on local health system contextual factors, such as specific caregiver knowledge gaps or staffing resources available at a health facility. Below we present our recommendations for implementors and policy-makers.

### Recommendation 1: when replicating immunisation interventions, the eight themes identified in this paper can inform adaptations to the local context

When replicating a specific intervention to another context, it is essential to consider the factors identified in our analysis, such as gender norms, the composition of the health workforce, the specific vaccine ecosystem of the country or sub-national region, routine vaccination policies and practices, dynamics between health workers and caregivers and the structure of the immunisation system and outreach processes. By incorporating these identified categories into preimplementation assessments, governments and implementers could better assess the extent to which an intervention is the right fit for a new context and the extent to which it needs to be tailored to fit the specific needs of the health system and population.

### Recommendation 2: countries should consider expanding or task shifting vaccination responsibilities to lower cadres of health workers

Expanding or task shifting vaccination responsibilities to lower cadres of health workers could improve service availability and reliability. This is supported by observed differences between Mozambique and Malawi, with our findings suggesting greater availability of services and stronger caregiver trust in Malawi’s large immunisation workforce of CHWs compared with Mozambique’s immunisation workforce of health workers who must balance multiple PHC roles. In addition, cocreated solutions in Mozambique emphasised the need to better leverage CHWs to strengthen immunisation services. This is further substantiated by evidence from studies in Burkina Faso, Malawi, Rwanda and Kenya that highlight how task shifting immunisation education, tracking and registering kids for vaccinations and in some cases, providing vaccination leads to increases in immunisation coverage and equity.^34^,^37^ These studies and our analysis suggest that this could positively impact caregivers’ immunisation experiences and improve access to reliable immunisation services.

### Recommendation 3: vaccinators should receive training and support on empathic communication

Experiences of poor treatment at health facilities were a common driver of vaccination dropout across Mozambique and Malawi. To address this, HCWs, CHWs and caregivers identified the need to improve interpersonal interactions between health workers and caregivers, especially in situations where a caregiver could be perceived as neglectful, such as bringing in their child late for vaccinations. Our findings show that HCWs and CHWs do not typically receive education or professional support related to interpersonal communication or cultivating empathy. This finding is consistent with other research that has shown the need for training the health workforce in empathetic communication methods as well as multichannel communication with caregivers can enhance vaccine uptake and acceptance.^3338^,^40^

### Recommendation 4: vaccine messaging needs to be tailored to the current vaccine ecosystem and answer practical information on vaccines

Our data suggest that in both Mozambique and Malawi there is a need for more comprehensive vaccine information that goes beyond general messages about vaccine importance, disease prevention and potential side effects. Vaccines messaging should include practical information that answers common questions and concerns caregivers typically have around vaccination. This includes providing readily available, up-to-date information on the vaccination schedule, new vaccine introductions, what to do if a vaccine dose is missed and how to replace vaccination cards. Additionally, vaccine messaging should be tailored to reach all members of a family, as our findings highlighted that when mothers have support from family members, including husbands, they are more likely to complete all of their child’s immunisations.

### Recommendation 5: provide clear public-facing communication on campaign timing and scope

Our findings suggest new vaccine introductions coupled with campaigns can contribute to confusion and increased hesitancy among caregivers. In these contexts, there is a clear need for enhanced and consistent public education around any changes to the vaccine schedule and/or increased frequency of vaccination campaigns. Additionally, when a new vaccine is introduced, all vaccine-related materials, including posters, vaccine cards and associated educational materials should be updated alongside the vaccine rollout. This ensures messaging remains clear and accurate, helping to mitigate any confusion or hesitancy.

### Strengths, limitations and future directions

The strengths of this analysis include the use of data generated from participatory qualitative methods, which elicited rich and nuanced descriptions of immunisation experiences and drivers of routine immunisation dropout. This enabled robust comparison across data collection sites and in relation to their respective health systems. Data were collected by caregiver researchers, who were from the same region and shared similar culture and language as participants and were able to build rapport and trust with caregiver participants, likely reducing the likelihood of social desirability bias and contributing to the overall trustworthiness of the data.

This analysis also had several limitations. These include potential selection bias due to different recruitment methods between study sites, which was a result of varying availability of vaccination registries and practices. Additionally, there were some challenges in recruiting individuals from certain religious and migrant groups. As a result, our findings may reflect experiences of participants who had slightly better access and higher trust in the public health system.

Another limitation is that data collection occurred 2 years apart during different phases of the COVID-19 pandemic, and slightly different tools were used across the two study sites. This may limit comparability of findings between Malawi and Mozambique. However, this was partially mitigated by asking specific questions in the interview guide about the impacts of the COVID-19 pandemic on routine immunisation, allowing us to compare responses across study sites. Further, while the solution ideas were generated by caregivers and health workers, EPI’s role in the prioritisation process may have impacted the degree to which these are considered ’bottom-up’ solutions, however, we believe EPI’s role in this process was critical to ensuring feasibility and sustainability of the proposed co-created solutions.

Despite these limitations, this study highlights opportunities for potential scalable interventions to address routine immunisation dropout such as task shifting immunisation responsibilities (education, coordination and vaccination) to lower cadres of health workers, expanding standard vaccine messaging to include practical information important to caregivers, tailoring vaccine messaging for both men and women and strengthening HCW and CHW education to include modules on interpersonal communication.

An important area for future research would be to explore the relative importance of the contextual and health system factors we identified, from the perspectives of both caregivers and health workers, in relation to routine immunisation dropout.

## Supplementary material

10.1136/bmjopen-2025-104490 online supplemental file 1

## Data Availability

Data are available on reasonable request.
