# Supplementary material for: Identifying context-specific drivers of routine childhood immunisation dropout in Mozambique and Malawi: a secondary thematic analysis of qualitative community-based participatory research data
Source: BMJ Open. 2025 Nov 19;15(11):e104490. doi: 10.1136/bmjopen-2025-104490 (PMC12636958; doi:10.1136/bmjopen-2025-104490)
Supplement: online supplemental file 1 [file bmjopen-15-11-s001.pdf]

Supplemental Table S1: Factors that may drive differences and similarities in immunization experiences

Contextual and Health System Factors	Malawi	Mozambique	Both
**Composition of the immunization workforce**	CHWs provide vaccinations. Caregivers perceived the workforce to be consistent, well-staffed, and trustworthy.	HCWs provide vaccinations along with other services, leading to feelings of overwork. Caregivers perceived high staff turnover, which eroded trust in the health system and immunization.	NA
**The Vaccine Ecosystem**	At the time of data collection, Malawi had a complex vaccine ecosystem consisting of new vaccine introductions, campaigns for outbreaks and COVID vaccines. This lead to confusion and vaccine hesitancy.	At the time of data collection, the vaccine ecosystem was relatively stable, and the COVID-19 vaccine was not yet introduced, leading to comparatively less hesitancy related to rumors and confusion.	Caregivers in both countries expressed lack of awareness of newly introduced MCV2 vaccine.
**Vaccination card policies**	In urban sites where there is no immunization registry (paper or electronic), caregivers who were missing their child's vaccination card	Mozambique maintains a paper record system. Participants reported that caregivers were sometimes denied vaccination for their child	Vaccination cards were recognized as key tools for remembering and keeping track of immunizations.

	were required to recall past vaccinations, and sometimes required to repeat all immunizations.<br><br>In rural areas, health workers referred to their paper records to fill out a new card or make a card on a piece of paper.	if they did not have a vaccination card.	
**Vaccination outreach models**	Health workers conducted multiple forms of outreach (outreach sites and door-to-door). Outreach services were perceived as reliable, frequent and contributed to improving access to vaccines in hard-to-reach areas.	Mobile brigades were cited as the most common kind of outreach, however they were reported to happen infrequently and with unknown times and locations. This contributed to challenges in accessing vaccination services.	Immunization outreach activities were cited as essential for improving access to vaccines, however they could be challenging to coordinate, and can be burdensome for HCW/CHWs to carry out.
**Gender Roles that Burdened Mothers**	NA	NA	Mothers are responsible for vaccination and if they are unable to take the child or are unsupported, it is less likely the child will get vaccinated. When fathers and other

			family members are actively involved in immunization, the chances of that child receiving all vaccines is improved.
**Limited Vaccine Information**	NA	NA	Caregivers and their families had knowledge gaps around the immunization schedule, vaccine safety and how to catch up on missed vaccines. Information is often shared through informal networks, like friends.
**Negative Health Worker Interactions**	NA	NA	Fear of being reprimanded or humiliated by health workers for either appearance or vaccination status led to hesitancy to return to the health facility.
**Pandemic Related Disruptions**	Data was collected after the COVID vaccine had been introduced, leading to reports of subsequent	NA	Pandemic related protocols such as masking, lockdowns, handwashing and

	hesitancy of routine immunizations.		suspended outreach led to disruptions in routine immunization services.
**Stockouts and resource constraints**			Disruptions in vaccine stock availability and other vaccine resources like cards, syringes etc. led to poor job satisfaction among health workers and disruption of services. Lack of resources for outreach was particularly pronounced. Disruptions in stock availability led to missed vaccines and reduced likeliness of caregivers returning for missed vaccines.
