# Supplementary material for: Identifying context-specific drivers of routine childhood immunisation dropout in Mozambique and Malawi: a secondary thematic analysis of qualitative community-based participatory research data
Source: BMJ Open. 2025 Nov 19;15(11):e104490. doi: 10.1136/bmjopen-2025-104490 (PMC12636958; doi:10.1136/bmjopen-2025-104490)
Supplement: Supplementary file 2 [file bmjopen-2025-104490.reviewer_comments.pdf]

PEER REVIEW HISTORY

BMJ Open publishes all reviews undertaken for accepted manuscripts. Reviewers are asked to complete a checklist review form and are provided with free text boxes to elaborate on their assessment. These free text comments are reproduced below.

ARTICLE DETAILS

Title (Provisional)

Identifying context-specific drivers of routine childhood immunization dropout in Mozambique and Malawi: a secondary thematic analysis of qualitative community based participatory research data

Authors

Lawrence, Emily; Metje, Alina; Matemba, Charles; Powelson, Jocelyn

---

VERSION 1 - REVIEW

**Reviewer**	**1**
**Name**	**Ray, Arindam**
**Affiliation**	**Bill and Melinda Gates Foundation India**
**Date**	**17-Aug-2025**
**COI**	**None**

Overall, the study is a well-conducted secondary qualitative analysis that provides valuable insights into immunization dropout drivers, though it has minor limitations in generalizability due to contextual and temporal differences between study sites.

**Reviewer**	**2**
**Name**	**Mirza, Imran**
**Affiliation**	**United Nations Childrens Fund UNICEF, Program**
**Date**	**01-Sep-2025**
**COI**	**None**

Congratulations on conducting this important study and submitting this manuscript. I was honored to review it and have given my comments in the attached document.

Overall Assessment

This is a valuable, well-structured manuscript that addresses an important and timely issue: the drivers of routine childhood immunization dropout in Malawi and

Mozambique. The use of community-based participatory research (CBPR), human-centered design (HCD), and cross-country comparative analysis provides rich insights into both shared and context-specific barriers. The findings are highly relevant for policymakers, program implementers, and global immunization partners.

That said, the paper would benefit from additional methodological clarification, more systematic cross-country comparisons, and a clearer articulation of actionable recommendations.

Major Comments

- Page 5, Line 5: The manuscript states that global childhood immunization coverage (DPT3) "stalled in 2023." In fact, coverage had been stagnant for nearly a decade before the pandemic. This requires correction for accuracy.
- Page 6, Line 6: The dropout rate in Malawi is cited as 33% (DPT1–MCV2). WHO/UNICEF estimates for 2024 report DPT1 coverage at 94% and MCV2 coverage at 69%, giving a dropout rate of ~27% rather than 33%. This should be rechecked and corrected.
- Page 8, Line 6: Data were collected two years apart (Mozambique: 2020–21, Malawi: 2022–23). Although mentioned as a limitation, the discussion should better account for contextual changes, particularly the COVID-19 pandemic. Lockdowns, service disruptions, and vaccine introductions may have introduced significant differences that shaped responses and experiences.
- Page 8, Line 30: Ideas generated in workshops were presented to EPI representatives and managers for selection. This may have biased outputs toward top-down solutions rather than those emerging directly from caregivers and frontline health workers. The implications of this should be explored and acknowledged.
- Page 9, Line 14: Different participatory tools were used (photovoice in Mozambique vs. photo-elicitation in Malawi with 37 pre-selected photos). This methodological difference could have influenced the type and depth of caregiver narratives but is not discussed. The impact of these differences warrants further reflection.
- Page 10, first paragraph: The roles of individual authors should not be described in the main text. This belongs in the "Author Contributions" section.

General Observations

- Results are presented descriptively but lack systematic synthesis. More explicit contrasts and theorization of differences between Malawi and Mozambique (e.g., CHW role, card policies, outreach strategies) would strengthen the analysis.

- Numerous themes are reported (gender roles, stockouts, card policies, health worker interactions), but their relative importance is unclear. Prioritizing 3–4 key drivers would sharpen the findings.
- The discussion restates results but could be better focused on actionable recommendations (e.g., policy reform on replacement cards, investment in CHW task-shifting, harmonization of campaign and routine immunization messaging).
- The innovative role of caregiver-researchers is commendable, but the potential for social desirability bias should be more fully explored. Similarly, VillageReach's dual role as implementer and researcher could have influenced participant responses and should be acknowledged.
- According to WHO Malawi and Mozambique 2024 DPT1 coverages are 94% and 90% respectively. While DPT3 coverages are 91% & 70%, and MCV2 are 69% and 44%. While delivery challenges in Mozambique as compared to Malawi has been mentioned, but not clear if during interviews with caregivers and health workers further probing was conducted that how DPT1 coverage is so high considering mentioned challenges. Considering high DPT1 coverage in both countries, seems vaccine demand within communities or access to services is not a major bottleneck. It's not clear if findings were investigated through this angle.

Recommendations for Authors

- **Methods:**
    - Expand the rationale for conducting secondary analysis across studies conducted at different times and with different tools.
    - Add a reflexivity statement on caregiver-researcher roles and VillageReach's dual position.
- **Results:**
    - Provide a clearer hierarchy of findings (e.g., via a thematic map).
    - Consider including basic counts to show how many participants raised each theme.
- **Discussion:**
    - Strengthen comparative analysis by explicitly linking findings to differences in health system structures.
    - Provide sharper, prioritized recommendations for policymakers and practitioners based on various drop-out drivers and bottlenecks identified.

- **Limitations:**
    - Expand on potential biases from recruitment challenges, methodological differences, and social desirability.
- **Presentation:**
    - Ensure consistent terminology ("dropout," "missed doses," "incomplete vaccination").
    - Streamline author roles into the contribution section.

Recommendation

Major Revision – This is a strong and relevant manuscript, but revisions are necessary to address methodological clarity, strengthen comparative insights, and sharpen the policy/practice implications.

VERSION 1 - AUTHOR RESPONSE

Reviewer 1:

Reviewer: 1
Dr. Arindam Ray, Bill and Melinda Gates Foundation India
Comments to the Author: Overall, the study is a well-conducted secondary qualitative analysis that provides valuable insights into immunization dropout drivers, though it has minor limitations in
generalizability due to contextual and temporal differences between study sites.

Response: Thank you for these comments and for your review, we greatly appreciate your reflection. We have addressed your comments about the limitations due to contextual and temporal differences in the discussion section (page 24, lines 18-330; page 25, lines 1-15).

Reviewer 2:

Reviewer: 2 Dr. Imran Mirza, United Nations Childrens Fund UNICEF

Comments to the Author: Congratulations on conducting this important study and submitting this manuscript. I was honored to review it and have given my comments in the attached document [SEE ATTACHED FILE].

Reviewer Overall Assessment: This is a valuable, well-structured manuscript that addresses an important and timely issue: the drivers of routine childhood immunization dropout in Malawi and Mozambique. The use of community-based participatory research (CBPR), human-centered design (HCD), and cross-country comparative analysis provides rich insights into both shared and context-

specific barriers. The findings are highly relevant for policymakers, program implementers, and global immunization partners. That said, the paper would benefit from additional methodological clarification, more systematic cross-country comparisons, and a clearer articulation of actionable recommendations.

<u>Response</u>: Thank you for your thoughtful and thorough review, this has certainly made for a stronger paper and analysis. Below we have responded to your comments and suggestions.

Reviewer Major Comments

1. **Page 5, Line 5**: The manuscript states that global childhood immunization coverage (DPT3) "stalled in 2023." In fact, coverage had been stagnant for nearly a decade before the pandemic. This requires correction for accuracy.

<u>Response</u>: We have revised the statement to clarify that the stalled coverage refers to the initial gains in immunization coverage seen after the pandemic, rather than stagnation that occurred before it. We have also added language referring to the stagnant immunization coverage from 2010-2019, as we agree this is important context for understanding the long-standing underlying factors that may be contributing to dropout rates globally. *We included an additional reference (1) to support this.*

2. Page 6, Line 6: The dropout rate in Malawi is cited as 33% (DPT1–MCV2). WHO/UNICEF estimates for 2024 report DPT1 coverage at 94% and MCV2 coverage at 69%, giving a dropout rate of ~27% rather than 33%. This should be rechecked and corrected.

Response: Thank you for catching this, we think what happened here is that we pulled from data accessed in early 2024 when we started drafting this manuscript. We have updated the data in the paper to reflect the 27% dropout rate (DPT1-MCV2) and updated to a more recent citation (18).

3. **Page 8, Line 6**: Data were collected two years apart (Mozambique: 2020–21, Malawi: 2022–23).

Although mentioned as a limitation, the discussion should better account for contextual changes, particularly the COVID-19 pandemic. Lockdowns, service disruptions, and vaccine introductions may have introduced significant differences that shaped responses and experiences.

<u>Response</u>: Thank you for this comment. Interestingly, this was one of the more surprising findings from our research. While challenges related to lockdowns and pandemic restrictions did come up in both data sets (Mozambique and Malawi), as this was a specific question we asked during interviews, they were not as prominent compared to other barriers. The barriers that were mentioned were actually quite similar across both countries, which is why we present them together in the results section under the theme "Pandemic Related Disruptions."

The main exception were differences introduced by the COVID-19 vaccine, which came up more frequently in our interviews in Malawi. We highlighted this as a specific difference in our results section. To make this clearer, we have now included a description of that difference in the newly added Table 3 (page 13).

4. **Page 8, Line 30**: Ideas generated in workshops were presented to EPI representatives and managers for selection. This may have biased outputs toward top-down solutions rather than those emerging directly from caregivers and frontline health workers. The implications of this should be explored and acknowledged.

<u>Response:</u> Thank you for this question and the opportunity to make this clearer in the manuscript. We would like to clarify that while the ideas generated during the workshop were presented to the EPI for selection to move into the prototyping phase, this occurred only after all ideas had been generated. These solution ideas came directly from the participating caregivers and health workers, ensuring a bottom-up approach. Their ideas were then shared with EPI managers for selection, in order to take feasibility and sustainability into account.

5. **Page 9, Line 14:** Different participatory tools were used (photovoice in Mozambique vs. photo- elicitation in Malawi with 37 pre-selected photos). This methodological difference could have influenced the type and depth of caregiver narratives but is not discussed. The impact of these differences warrants further reflection.

<u>Response</u>: Thank you for this comment. We have included this as a limitation of the study in the discussion section (page 24, lines 27–29). The data from both sets of interviews were similar in depth, largely due to the use of open-ended probing throughout the interviews and the inclusion of similar questions following the photovoice and photo-elicitation portions. While we had hoped to conduct photovoice in Malawi as well, however we opted for the photo-elicitation method due to financial and time constraint

6. **Page 10,** first paragraph: The roles of individual authors should not be described in the main text. This belongs in the “Author Contributions” section.

<u>Response</u>: Thank you, we have removed reference to author roles for improved flow of the manuscript.

Reviewer 2 General Observations

1. Results are presented descriptively but lack systematic synthesis. More explicit contrasts and theorization of differences between Malawi and Mozambique (e.g., CHW role, card policies, outreach strategies) would strengthen the analysis.

<u>Response</u>: Thank you for this comment. We have addressed this by adding a table (Table 3; pages 10-14) to better synthesize the similarities and differences between Malawi and Mozambique. We attempted to address this in the discussion

2. Numerous themes are reported (gender roles, stockouts, card policies, health worker interactions), but their relative importance is unclear. Prioritizing 3–4 key drivers would sharpen the findings. The discussion restates results but could be better focused on actionable recommendations (e.g., policy reform on replacement cards, investment in CHW task-shifting, harmonization of campaign and routine immunization messaging).

<u>Response:</u> Thank you for this thoughtful comment on our results can be more clearly and concisely reported. As with our response to the above comment, we have created a table to more clearly summarize key themes (Table 3; pages 10-14). We have also improved our discussion by sharpening the recommendations to be more specific and numbered.

3. The innovative role of caregiver-researchers is commendable, but the potential for social desirability bias should be more fully explored. Similarly, VillageReach's dual role as implementer and researcher could have influenced participant responses and should be acknowledged.

Response: Thank you for this comment as it's something we considered carefully throughout the study. We believe the caregiver researcher role helped minimize social desirability bias. In both Malawi and Mozambique, our past work has shown that participants often give responses they think an authority figure wants to hear. To address this, we intentionally selected peers as researchers to create a more comfortable environment. We additionally incorporated self-reflection exercises into the caregiver researcher training and held weekly debriefs to reflect on how researchers and VillageReach were perceived by communities and participants in order to further mitigate any potential social desirability bias.

Caregiver researchers shared that their peer status helped build rapport and trust, which they felt allowed for more open and honest conversations. Their reflections can be accessed here.

Regarding VillageReach's dual role as implementer and researcher, we agree this is important to acknowledge. We did however find that this dual role helped build trust and shared ownership with communities, health workers, and government partners. This enabled smoother implementation and stronger alignment with community-identified priorities. One ongoing challenge was ensuring that solutions were grounded in community voices rather than researcher interpretation. We addressed this by closely collaborating with caregiver researchers during the solution development and implementation phase.

To further reduce potential bias, we partnered with the University of the Western Cape and the University of Cape Town to conduct external evaluations in both countries. Their assessments found that the CBPR and HCD approach produced findings that closely reflected participants' lived experiences. These results were published in BMC Research Involvement and Engagement and can be accessed here. We have included a discussion of these strengths and limitations in the manuscript (page 24, line 18-29).

4. According to WHO Malawi and Mozambique 2024 DPT1 coverages are 94% and 90% respectively. While DPT3 coverages are 91% & 70%, and MCV2 are 69% and 44%. While delivery challenges in Mozambique as compared to Malawi has been mentioned, but not clear if during interviews with caregivers and health workers further probing was conducted that how DPT1 coverage is so high considering mentioned challenges. Considering high DPT1 coverage in both countries, seems vaccine demand within communities or access to services is not a major bottleneck. It's not clear if findings were investigated through this angle.

Response: Thank you for this comment. The original studies and this analysis aimed to understand the drivers of dropout in routine immunization. Our focus was less on why DPT1 coverage was high and more on what factors contributed to children dropping off the schedule later. While we didn't explicitly investigate the reasons behind the strong DPT1 coverage, our interviews did explore what changed in caregivers' circumstances or experiences that led to dropout later in the schedule.

Our findings show that both demand and access continue to play a role throughout the immunization journey. We do not view these factors as fixed; rather, they are dynamic and responsive to the broader environment. Shifts such as new vaccine introductions, outbreaks, campaigns, or even household-level changes (like financial strain or shifting family support) all influenced whether children stayed on schedule.

These dynamics operate throughout the immunization journey and can be shaped by household, health system, and national factors. While some of these may also affect zero-dose children, we believe the underlying barriers in those contexts are likely different and were outside the scope of this analysis.

Recommendations for Authors

1. **Methods**:
    a. Expand the rationale for conducting secondary analysis across studies conducted at different times and with different tools.

Response: We have added a paragraph to provider further explanation for the gap in data collection between the two sites, which was the result of a follow-up grant to expand the study to Malawi and conduct this secondary analysis. (pg 6 lines 13-16, pag 7 lines 1-3). Added text reads: "An initial grant supported research from February 2020 to March 2021 in Gile and Namarroi Districts in Zambézia Province, Mozambique. A follow- on grant then expanded this research to Lilongwe and Mzimba North Districts in Malawi from July 2022 to February 2023. The second grant expanded the scope of the study in three ways: it added an urban site to enable urban–rural comparisons; supported cross- country analyses of similarities and differences between findings in Malawi and Mozambique; and examined which interventions could be scaled broadly versus which required context-specific adaptation to strengthen full immunization coverage."

    b. Add a reflexivity statement on caregiver-researcher roles and VillageReach's dual position.

Response: Thank you, we have added a reffexivity statement on page 7, lines 1c-22. The text reads: "The research team consisted of local, national and international researchers with diverse disciplinary, linguistic, and cultural backgrounds. To mitigate bias and strengthen reffexivity, all researchers participated in regular debriefs during both data collection and analysis. These sessions provided space to reffect on how their lived experiences and professional backgrounds may be shaping the ways data were collected, interpreted and understood. Data interpretation was done collaboratively, and to help ensure the validity of the findings, preliminary results were shared with participating health facilities and community representatives for feedback and validation."

2. **Results**:
    a. Provide a clearer hierarchy of findings (e.g., via a thematic map).

Response: Thank you for your comment on how to improve the clarity of our findings. While we are unable to provide a hierarchy of findings, as this is not consistent with the design of our study or the structure of the interview guide we used, it is also important to note that participants often mentioned multiple factors or themes that interacted with each other rather than identifying a single issue. To address your suggestion and improve clarity, we have included a summary table of the findings to help readers better follow the analysis. Additionally, we agree that your idea of identifying a hierarchy would be valuable and think it could be the focus of a follow-up quantitative study. Now that we have identified the key health system and contextual factors contributing to dropout, a quantitative study could examine the

prevalence of each factor. This would help governments and policymakers prioritize which issues are most important to address.

b. Consider including basic counts to show how many participants raised each theme.

Response: Thank you for the suggestion. While we understand the interest in seeing how many participants contributed to each theme, this isn't possible to include in the paper. Because we conducted a thematic analysis rather than a content analysis, we did not track the frequency of themes across participants. Our approach focused on identifying patterns of meaning rather than quantifying responses, and we did not code the data by theme. Instead, our coding informed the development of themes, often with multiple codes contributing to a single theme and some participants referencing the same code multiple times. We have aimed instead to ensure that our findings are well supported with representative quotes and detailed descriptions of themes.

3. **Discussion**:
    a. Strengthen comparative analysis by explicitly linking findings to differences in health system structures.

Response: Thank you for this comment, we addressed this by adding in Table 3 which more clearly showcases similarities and differences between Malawi and Mozambique findings.

b. Provide sharper, prioritized recommendations for policymakers and practitioners based on various drop-out drivers and bottlenecks identified.

c.

Response: Thank you for this comment as we feel it has improved the clarity of our discussion and recommendations. We have addressed this by sharpening each recommendation and numbering them into 5 key recommendations in the discussion section.

4. **Limitations**:
    a. Expand on potential biases from recruitment challenges, methodological differences, and social desirability.

Response: We have expanded on the limitations, including recruitment challenges and methodological differences in the discussion section (page 24, lines 18-330; page 25, lines 1-15).

5. **Presentation**:
    a. Ensure consistent terminology ("dropout," "missed doses," "incomplete vaccination").

Response: Thank you. For the term missed doses, we are using that as a past participle adjective here as this is in context to caregiver reactions on what they should do when they miss a dose as opposed to as a noun as we have used it throughout the paper. We have updated that reference of missed doses to missed vaccinations in order to be consistent with the rest of the paper.

b. Streamline author roles into the contribution section.

Response: We have removed reference to author roles on page S lines 10-17 for improved ffow of the manuscript.

VERSION 2 - REVIEW

**Reviewer**	**2**
**Name**	**Mirza, Imran**
**Affiliation**	**United Nations Childrens Fund UNICEF, Program**
**Date**	**11-Oct-2025**
**COI**	

Thank you for your thoughtful and comprehensive revisions to the manuscript. It is clear that you have carefully considered and responded to each of the comments with great diligence. The revised version demonstrates notable improvements in clarity, methodological transparency, and policy relevance.

Overall, I am pleased to see that the majority of the earlier concerns have been fully addressed. The manuscript is now substantially strengthened, and it makes a valuable contribution to the literature on immunization dropout, community-based participatory methods, and comparative qualitative inquiry in sub-Saharan Africa.

Below is a brief summary of my overall assessment of the revised submission:

The authors have clearly integrated the suggested revisions. Key improvements include:

• Correction of statistical inaccuracies and updated data sources (e.g., DPT1–MCV2 dropout rates).

• Substantial enhancement of the Discussion section, including clearer cross-country comparisons and actionable policy recommendations.

• Addition of a reflexivity statement, which greatly improves methodological transparency.

• Expansion of the Limitations section to include recruitment challenges, methodological differences, and potential biases.

• Streamlined presentation and consistent terminology throughout.

These revisions have resulted in a more coherent and analytically rich paper that is accessible to both researchers and program implementers.

Remaining Minor Clarifications:

Only a few points may benefit from minor clarification or further emphasis:

• The manuscript now clearly explains that community-generated ideas were reviewed by EPI managers to ensure feasibility; it would be valuable to add one brief line acknowledging that this process may also have influenced prioritization (which you have already noted constructively in your response letter).

• While it is understandable that your qualitative design does not permit quantification or ranking of themes, your explanation in the Discussion—highlighting that future quantitative studies could estimate relative importance—is appropriate and sufficient.

• The clarification that your study focuses on dropout rather than initial uptake is well phrased and aligns with the scope of the analysis.

These are very minor editorial points, and the manuscript is otherwise complete and well-presented.

Overall Assessment:

The paper now meets the standards for publication in BMJ Open. It presents a compelling and contextually grounded examination of immunization dropout through participatory research methods. The authors' integration of cross-country insights, combined with practical policy recommendations, makes this a timely and policy-relevant contribution.

I commend the authors for their thoughtful revisions and the clarity of their responses. The paper is now ready for publication, pending any final minor editorial adjustments by the journal.

With appreciation for your excellent work and responsiveness,

---

VERSION 2 - AUTHOR RESPONSE

Reviewer: 2 Overall Assessment of Revisions
Dr. Imran Mirza, United Nations Childrens Fund UNICEF

Comments to the Author:
Thank you for your thoughtful and comprehensive revisions to the manuscript. It is clear that you have carefully considered and responded to each of the comments with great diligence. The revised version demonstrates notable improvements in clarity, methodological transparency, and policy relevance.

Overall, I am pleased to see that the majority of the earlier concerns have been fully addressed. The manuscript is now substantially strengthened, and it makes a valuable contribution to the literature on immunization dropout, community-based participatory methods, and comparative qualitative

inquiry in sub-Saharan Africa.

Below is a brief summary of my overall assessment of the revised submission:

The authors have clearly integrated the suggested revisions. Key improvements include:

- Correction of statistical inaccuracies and updated data sources (e.g., DPT1–MCV2 dropout rates).
- Substantial enhancement of the Discussion section, including clearer cross-country comparisons and actionable policy recommendations.
- Addition of a reflexivity statement, which greatly improves methodological transparency.
- Expansion of the Limitations section to include recruitment challenges, methodological differences, and potential biases.
- Streamlined presentation and consistent terminology throughout.

These revisions have resulted in a more coherent and analytically rich paper that is accessible to both researchers and program implementers.

Remaining Minor Clarifications:
Only a few points may benefit from minor clarification or further emphasis:

- The manuscript now clearly explains that community-generated ideas were reviewed by EPI managers to ensure feasibility; it would be valuable to add one brief line acknowledging that this process may also have influenced prioritization (which you have already noted constructively in your response letter).
- While it is understandable that your qualitative design does not permit quantification or ranking of themes, your explanation in the Discussion—highlighting that future quantitative studies could estimate relative importance—is appropriate and sufficient.
- The clarification that your study focuses on dropout rather than initial uptake is well phrased and aligns with the scope of the analysis.

These are very minor editorial points, and the manuscript is otherwise complete and well-presented.

<u>Overall Assessment:</u>
The paper now meets the standards for publication in BMJ Open. It presents a compelling and contextually grounded examination of immunization dropout through participatory research methods. The authors' integration of cross-country insights, combined with practical policy recommendations, makes this a timely and policy-relevant contribution.

I commend the authors for their thoughtful revisions and the clarity of their responses. The paper is now ready for publication, pending any final minor editorial adjustments by the journal.
With appreciation for your excellent work and responsiveness.

Responses to Reviewer Comments

1. The manuscript now clearly explains that community-generated ideas were reviewed by EPI managers to ensure feasibility; it would be valuable to add one brief line acknowledging that

this process may also have influenced prioritization (which you have already noted constructively in your response letter).
Response: Thank you for this comment. We have added in acknowledgement that this process may have influenced solution outcomes in the discussion section on page 20, lines 28-32.

2. While it is understandable that your qualitative design does not permit quantification or ranking of themes, your explanation in the Discussion—highlighting that future quantitative studies could estimate relative importance—is appropriate and sufficient.

   Response: Thank you for this comment and we do hope that this work inspires a follow-up study exploring the relative importance of these factors as it could have a powerful influence on policy.

3. The clarification that your study focuses on dropout rather than initial uptake is well phrased and aligns with the scope of the analysis.

   Response: Thank you for this acknowledgement and for the original thoughtful comment.
